# Neural Network Methods in the Development of MEMS Sensors

**DOI:** 10.3390/mi15111368

**Published:** 2024-11-12

**Authors:** Yan Liu, Mingda Ping, Jizhou Han, Xiang Cheng, Hongbo Qin, Weidong Wang

**Affiliations:** 1 School of Mechano-Electronic Engineering, Xidian University, Xi’an 710071, China; 23041212735@stu.xidian.edu.cn (M.P.); 22041212665@stu.xidian.edu.cn (J.H.); 22041212669@stu.xidian.edu.cn (X.C.); qhb0920qhb@xidian.edu.cn (H.Q.); 2 CityU-Xidian Joint Laboratory of Micro/Nano Manufacturing, Shenzhen 518057, China

**Keywords:** MEMS sensor, neural network, structural design, fabrication, compensation, calibration

## Abstract

As a kind of long-term favorable device, the microelectromechanical system (MEMS) sensor has become a powerful dominator in the detection applications of commercial and industrial areas. There have been a series of mature solutions to address the possible issues in device design, optimization, fabrication, and output processing. The recent involvement of neural networks (NNs) has provided a new paradigm for the development of MEMS sensors and greatly accelerated the research cycle of high-performance devices. In this paper, we present an overview of the progress, applications, and prospects of NN methods in the development of MEMS sensors. The superiority of leveraging NN methods in structural design, device fabrication, and output compensation/calibration is reviewed and discussed to illustrate how NNs have reformed the development of MEMS sensors. Relevant issues in the usage of NNs, such as available models, dataset construction, and parameter optimization, are presented. Many application scenarios have demonstrated that NN methods can enhance the speed of predicting device performance, rapidly generate device-on-demand solutions, and establish more accurate calibration and compensation models. Along with the improvement in research efficiency, there are also several critical challenges that need further exploration in this area.

## 1. Introduction

The sensor is one of the essential components of modern systems, which can provide abundant original data for information collection, strategic decisions, and intelligent responses. With the evolution in recent decades, the MEMS sensor has taken up the predominant position in sensing devices [1]. This technology offers a compact and lightweight solution for the measurement of physical, chemical, and biological parameters and has found significant applications in consumer electronics, the automobile industry, IoT, healthcare, environment monitoring, etc. [2,3,4,5]. Considering the sensing mechanism, a series of electrical parameters, including resistance, capacitance, inductance, and electric charge, are often utilized as the medium to transform measurands into accessible digital/analog signals. The vigorous development of the MEMS sensor in design, fabrication, and characterization has become a significant booster for modern society.

In recent years, sophisticated applications have continuously pursued more and more specific features from MEMS sensors, posing higher requirements for the construction scheme. Consequently, conventional approaches often fall short of the capacity for revolutionary improvement, necessitating the continuous proposal of innovative solutions. Taking the piezoresistive pressure sensor as an example, the initial design utilized a flat diaphragm to capture the fluid pressure [6,7]. However, as the measurement range decreased, the flat-diaphragm structure could not achieve a favorable compatibility between sensitivity and linearity, failing to fully complete the low-pressure testing task. Therefore, the researchers introduce grooves, bosses, peninsular beams, and paddles into the diaphragm to produce stress concentration and partial stiffness in the diaphragm, which is anticipated when promoting sensitivity and diminishing nonlinearity [8,9,10,11,12]. Despite the continuous technological innovations in sensor structure, the increased complexity also causes great burdens in theoretical modeling, finite element simulation, and parameter optimization [13,14]. In this scenario, the traditional approaches relying on experience and iterative trial-and-error are becoming inefficient in the design and optimization of advanced sensor structures [15].

The fabrication and characterization of MEMS sensors also face challenges from the demand for high-end devices. It is necessary to enhance the processing capability for realizing diverse tools and detecting capability to identify the possible defects in wafers or devices. The enhancement of data quality in device characterization requires extra hardware/software modules to achieve decoupling, noise reduction, and drift compensation. The fulfilling of these tasks also depends on the high-precision models to describe the involved interrelationships, specialized skills, significant efforts, and prolonged attempts.

As a result, there is a growing need for more efficient design, modeling, and optimization techniques that can leverage the latest computational tools and algorithms to achieve on-demand design/processing capabilities. The last decade has seen a surge in commercial applications utilizing artificial intelligence (AI) technology, which has delivered groundbreaking achievements that are revolutionizing the way we live [16,17]. The use of large AI models will transform our approach to information search and generation [18,19]. As the core components of current AI technology, the neural network (NN), with its excellent regression and classification capabilities, has solved a multitude of issues in large-scale data processing, pattern recognition, and performance prediction, becoming a powerful computational tool in scientific research [20], medicine [21,22,23], agriculture [24,25,26], and engineering [27,28,29]. Accordingly, the availability of computational resources, coupled with the maturation of NN methods, has profoundly impacted the development of sensor technology. NNs have offered new opportunities to address the challenges in developing advanced MEMS sensors. Researchers have employed various neural network models during the design phase to predict device performance, optimize structural dimensions, and generate structural topology. The decoupling, compensation, and denoising of output data also receive favorable promotions. These efforts have made significant contributions to enhancing sensor performance, indicating the fact that NN has become a game changer in MEMS sensor research.

Along with the emerging achievements in applying NN methods to the development of MEMS sensors, several review works have been made and published, summarizing the roles of NN in the development of photoelectric sensors [30], gas sensors [31,32], microwave sensors [33,34], inertial sensor [35], and biosensors [36]. Some work has also discussed the intelligentization of MEMS sensors triggered by the ability of intelligent methods to obtain interested information from output datasets [37,38]. In the meantime, a few reviews refer to the possible applications of NN in the design, calibration, or compensation of MEMS sensors, and the perspective on future design paradigms is also discussed [39,40,41]. However, these papers have not provided a systematic and in-depth insight into the roles played by NN technology throughout the entire development process of MEMS sensors, and the latest studies have not been effectively demonstrated. This paper will successively introduce the neural network technologies available in the design, manufacturing, and output signal processing of MEMS sensors, focusing on the advantages of this new research model. It should be noted that this paper mainly emphasizes the achievements pertinent to sensor functionality and performance enhancement, and the works about further learning from the processed output signals for certain applications are out of the scope.

In the following parts, this paper will be divided into three modules: the first module, namely Section 2, provides a brief overview of the development of MEMS sensors and compares the difference between traditional and NN-based developing approaches; the second module sequentially discusses the application of NN methods in sensor design (Section 3), fabrication (Section 4), and output processing (Section 5); the last part discusses the challenges and prospects for this domain and provides a brief conclusion for this paper.

## 2. Overview of the Development of MEMS Sensors

**Figure 1:**
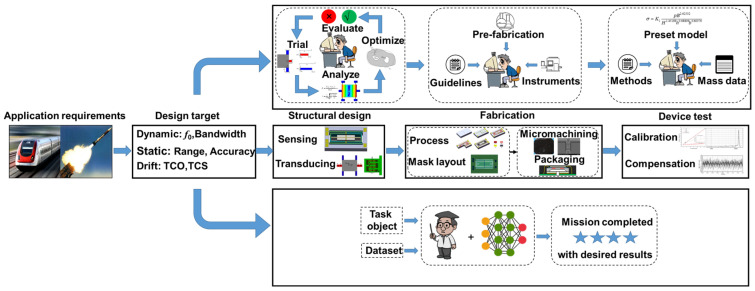
Overview of a single iteration in the development of MEMS sensors with conventional and NN methods.

Over the past few decades, MEMS technology has opened new avenues in developing high-performance sensors for various applications. MEMS sensors were first proposed in the 1960s and progressively scaled up since the 1980s, following the research and development in integrated circuit (IC) technology [42]. Additionally, their manufacturing techniques also inherit the photolithography of ICs, and the MEMS sensors must adhere to a boundary that the device structures can be defined and manufactured according to the two-dimensional layouts. Taking the physical sensor as an example, the development of MEMS sensors typically includes the following steps (as shown in the middle of Figure 1):

(1) Structural design, mainly including clarification of design requirement, selection of sensing mechanism, design of sensing structure and arrangement of transducing elements. Firstly, the desired dynamic and static criteria should be extracted from the applications. Considering the form of the output signal, a proper sensing mechanism is then selected from piezoresistive, capacitive, piezoelectric, or resonant ways. The design of the sensing structure should consider the required dynamic (e.g., resonant frequency, bandwidth) and static (e.g., range, sensitivity, linearity, repeatability, etc.) performances [43]. Concerning the working mode of different sensing mechanisms, fundamental structural elements (e.g., beam, membrane, plate, and block) are combined together with specific dimensions and locations to effectively capture the targets, where the manufacturability should be treated as the foundation [44]. Lastly, the transducing elements, e.g., piezoresistor, capacitor, piezoelectric element, and resonator, are introduced to fulfill the conversion of mechanical parameters in the sensing structure to the output signals of the sensor [45,46,47].

(2) Device fabrication. Adhering to the guidelines, the process procedure for fabrication is determined with mask layouts for photolithography and the main parameters for implementation. The sensor chip is micromachined by several processes and packaged after necessary wafer-level and device-level quality inspections [48].

(3) Device test. It is the final step before the sensor reaches the retail shelf, which mainly conducts the output compensation and calibration. A comprehensive characterization of the sensor prototypes is conducted to ascertain their dynamic and static parameters and compensation strategies are proposed to mitigate the output drifts induced by noise, temperature, and humidity. Thereby, a serviceable and robust sensor product is ready for listing.

As shown in the upper part of Figure 1, the conventional heuristic approaches for sensor design can be regarded as a trial-and-error matter, which largely depends on the knowledge, experience, and available tools and often requires a significant investment of time and resources [49]. Researchers first generate a possible concept with their personal knowledge and validate its features through mathematical or statistical methods to adjust the topology and dimensions. Some optimization methods, such as particle swarm optimization (PSO) [50,51], genetic algorithm (GA) [52,53], and gradient-based methods [54], are utilized to accelerate the design process. During this, inappropriate solutions are continuously identified and discarded, and a significant amount of time and resources are consumed in the pursuit of optimized solutions. Moreover, the initial unsuitable scheme induced by the understanding limitation will further worsen the situation. In the fabrication of MEMS sensors, the determination of process parameters requires a combination of empirical and accompanying wafer validation. For the pivotal and difficulty steps, a pre-fabrication verification for procedure and parameter refinements is more critical. The detection of fabrication defects also requires the investment of manpower. The calibration and compensation of MEMS sensors mainly depend on the pre-established empirical model, and the feature parameters are derived and modified from the combination of this model and experimental results. Obviously, the deviation of empirical model can significantly impair the processing performance, which is also a difficult work for the conventional treatments. Furthermore, the illustration merely depicts a single iteration within the research cycle. It is likely that numerous full-cycle validations are necessary throughout the MEMS sensor development process to culminate in the final product. Despite rapid progress has been made in the development of MEMS sensors, great challenges still lie in conventional researching methods. New developing approaches are critically needed to fulfill the task of providing schemes on-demand.

The introduction of NN methods in recent years provides a practical path for accelerating the development and improving the performance (down in Figure 1). Benefiting from the capability to learn and extract high-level features from large-scale data, the NN methods can obtain convoluted correlations between various parameters to lead researchers toward the optimized goals in sensor development. These advantages are being demonstrated in the development of MEMS sensors. Researchers can use trained neural networks to predict the design performance of a device configuration, and a practical structural solution can also be generated in real-time according to the required performance; defects in the fabrication can be identified through machine vision methods; the interference information in output signals can also be effectively identified and separated, thereby establishing a more accurate model to describe the correspondence between input measurands and output signals. The NN methods have presented an important opportunity for researchers to think with a more powerful and unconstrained style about the entity and data of MEMS sensors. It should be noted that AI is about creating machines or systems capable of performing tasks that typically require human intelligence, encompassing a wide range of content and technologies. This article primarily focuses on the application of learning technologies based on neural networks in the development of MEMS sensors, and the benefits of combining other intelligent methods are also mentioned. The following sections will provide detailed descriptions of the NN methods in the development of MEMS sensors.

## 3. NN Methods in the Design of MEMS Sensors

The structural design of MEMS sensors mainly focuses on the sensing structure and accompanying transduction elements. Generally, the arrangement of transduction elements is determined after choosing the proper sensing mechanism and structural configuration, which is commonly finished through a routine procedure. Thus, the design of the sensing structure will be expressed in detail, dealing with the issues in structural geometry and dimension.

### 3.1. NN Methods in Dimension Optimization

Ideally, the NNs in dimension optimization can be regarded as a generator to quickly produce size parameters that can meet the measuring requirements. This work can be divided into four steps: (1) the key performances and target dimensions are clarified; (2) a dataset, containing the correlation between performances and dimensions, is established for NN training, validation, and test; (3) a proper NN model is constructed, trained and tested to map the correlations in dataset and predict the device performance under a certain size combination; (4) the trained NN plays the function of generator to quickly provide practical dimensions according to the required performance. It is not necessary to fully execute all these operations in every design, and the role of NNs also varies within different works.

**Figure 2:**
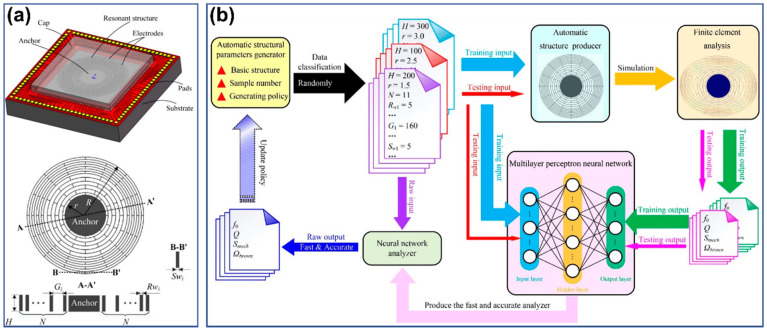
The disk MEMS resonator for dimensional optimization with NN method: (**a**) the structural diagram (**up**) and target dimensions (**down**), (**b**) the architecture and operating process of simulation analyzer based on MLPNN. Reproduced under the terms and conditions of the Creative Commons Attribution license of [55].

The disk MEMS resonator (DMR) in Figure 2a is taken as an example to show the operation of establishing a dimension–performance model using the NN method [55]. Six structural parameters, including structural thickness *H*, anchor radius *r*, ring number *N*, ring thickness *R_w_*, ring gap *G*, and spoke width *S_w_*, feature a complicated correlation with the four core performance indicators, including fundamental frequency *f*_0_, quality factor *Q*, mechanical sensitivity *S*, mechanical thermal noise Ω. Figure 2b shows the process of using a multilayer perceptron neural network (MLPNN) to correlate the structural parameters and performance indicators. Firstly, DMRs with random dimensions are produced by an automatic generator and then passed to the finite element analysis (FEA) tool for characteristic simulation. A dataset with 4000 groups of correlating data is produced, among which 3200 sets are randomly chosen as the training set, and the remaining 800 sets are for testing. In the training phase, the dimensional parameters of MDRs are treated as the input, and the corresponding performance indicators are treated as the output. After a 516 min training on GPU (NVIDIA GeForce GTX 1660Ti, Santa Clara, CA, USA), a predicting model for the performances under different dimensions is obtained with an error <6% (compared to the FEA results). In terms of predicting speed, the NN method only consumes 1/15,000 of FEA time (0.033 min vs. 496 min for 200 samples). Although a certain amount of time is consumed by the preparation of datasets, the NN methods for DMR prediction still exhibit significant advantages to support the requirements of design on-demand. Similarly, the back propagation neural network (BPNN) can also implement the abovementioned modeling, and the speed is several orders of magnitude faster than FEA with a regression accuracy ≥96% [56].

**Figure 3:**
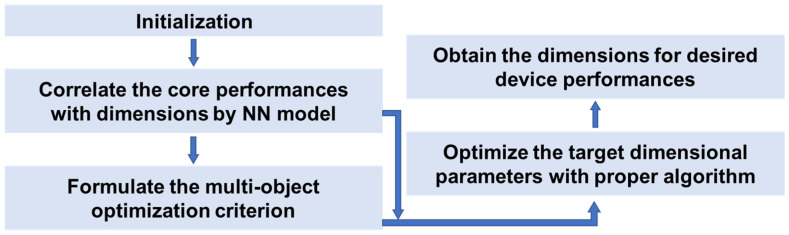
Flowchart of the optimization strategy using NN-based correlation models.

Then, it is necessary to fully leverage the obtained NN-based correlation models to optimize the structural dimensions according to the required performances. Herein, a simple scheme uses the trained NN model as a part of the optimization to replace the theoretical functions or FEA in conventional methods. Fei’s group has conducted a series of optimizing works for piezoelectric ultrasonic transducers with the help of NN methods, whose typical procedure can be shown in Figure 3 [57,58,59]. Firstly, the performance indicators, such as the electromechanical coupling coefficient, center frequency, bandwidth, and pulse width, are correlated to the core dimensions by NN. Then, an optimization algorithm (e.g., PSO) is conducted based on the preset multi-objective optimality criteria, where the NN is used to evaluate the deviation between iteration results and desired ones. The validating results demonstrate that the performance parameters from the developed strategy are in good agreement with FEA and experimental ones. Moreover, the GA algorithm can be utilized to set the initial weights of the NN model to enhance its convergence toward the global optimal solution [58].

**Figure 4:**
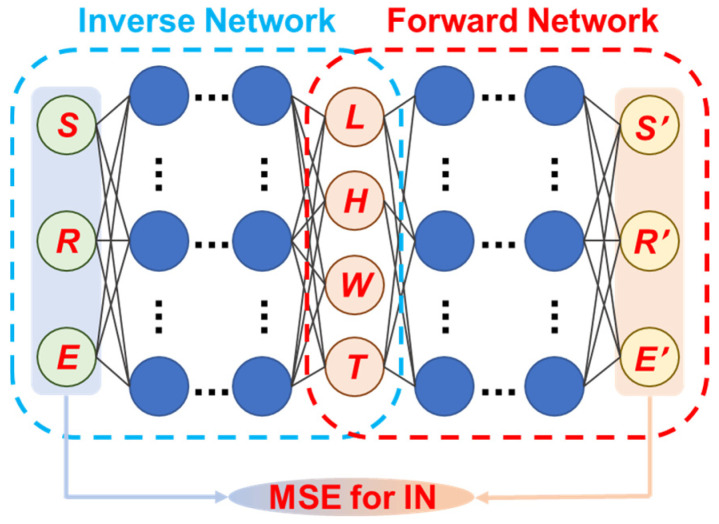
Sketch for the tandem network constructed by inverse network (IN) and pre-trained forward network.

However, the NNs in these abovementioned works still do not function as a dimension generator, which can directly produce dimensions according to the input performances (namely, the reverse model of these predictive ones). This proposal may suffer from the issue that a certain performance can be achieved by different dimensions, which may bring out the one-to-many mapping non-uniqueness problem in the training of reverse NNs and make them converge with a large error [60,61]. To address this issue, Zhang et al. merge a forward prediction model with a reverse generation model to create a tandem network that can rapidly output the corresponding geometries for the sensing diaphragm in MEMS capacitive pressure sensors [62]. As shown in Figure 4, the tandem network uses a reverse network to map from performances to size and a forward network to map from size to performances, and the output of the reverse network is subsequently input to the latter. The forward network is independently trained based on the FEA dataset. The weights of the reverse network are then updated according to the deviation between its inputs and the performance predicted by the forward network with the dimensions from the reverse network. After necessary validation and testing, the whole tandem network can fulfill the function of the dimension generator. In the task for a square sensing diaphragm, the tandem network can achieve an error of 7.05 × 10^−6^ after 9.2 × 10^3^ epochs in the measuring range, sensitivity, and linearity. In contrast, the error of a single inverse network can be up to 3.71 × 10^−2^, demonstrating the superiority of this new network model. Additionally, FEA results show that the generated diaphragm can achieve all these target performances with an error of <0.91%. Considering the generating speed, the time required by a tandem network is only 3 ms, while the time consumed by traditional FEM can be up to 10^3^ s. This tandem network approach features high accuracy and versatility, high design efficiency, and low computational cost.

### 3.2. NN Methods in Geometrical Design

The application of NN methods in geometrical design for MEMS sensors can be a more promising frontier work. Traditional geometrical design often employs evolutionary iteration or topology optimization methods to generate structural solutions that can meet a certain performance requirement [63,64]. These tasks often require extensive iterative calculations or demand specialized expertise, posing a significant burden for the designers. In the meantime, many of their results cannot be well compatible with the operating and micromachining mechanisms of MEMS sensors [54,65,66,67,68,69]. These situations provide a strong motivation for applying NN methods in the geometrical design of MEMS sensors, but the implementations are much more complicated than the works for dimension.

**Figure 5:**
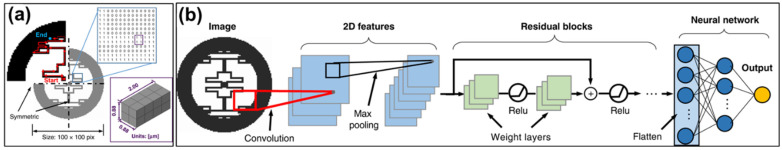
The structure for the ResNet model in predicting performances of resonator: (**a**) 100 × 100 pixelated binary 2D image to present the resonator; (**b**) structure of the ResNet model. Reproduced under the terms and conditions of the Creative Commons Attribution License of [71].

Lin’s group from UC Berkeley has constructed a practical approach for generating MEMS geometries with NNs, and several validations on MEMS resonators have been conducted. Similar to the dimensional issues, the first work is to determine the correlation between structural configuration and device performance. Inspired by the fact that MEMS devices are mostly manufactured based on two-dimensional patterns, 100 × 100 pixelated binary 2D images are used to represent the resonator body, where the black portions for the solid structures with a preset thickness and white portions for voids. An effective depth-first search algorithm is built to generate the geometries and guarantee continuity. The candidate images are sent to FEA for the variables of interest. Then, a ResNet model is utilized to connect the labeled FEA results with input 2D images. The structures of the used strategy can be found in Figure 5. Taking the circular disk resonator, for instance, the ResNet, after a 300 min training, can predict the first four resonant frequencies of 1000 candidates within 0.18 min, which is 4000 times faster than FEA with an accuracy >95% [70,71]. This predicting work provides a foundation for the subsequent generation toward specific design goals.

Furthermore, some iterative methods are combined with the pixelated binary 2D images. For instance, GA can progressively alter the black-and-white status of certain areas to pursue a new configuration for the target MEMS device. This method has been used in the design of weakly coupled resonators, and the obtained final coupling factors are lower than 98% of the initial random designs within 200 generations [72]. In the case study of circular-shaped MEMS resonators, a conditional generative adversarial network (CGAN) is used to fulfill the systematic MEMS structure design task [73]. The generator ***G*** and discriminator ***D*** form a standard GAN and are updated alternately to ensure the geometrical similarities between training samples and newly generated candidates. The predictor ***Q*** is a pre-trained network that maps the geometries with physical properties. ***G*** generates the geometry candidate, and ***D*** provides the vector that indicates the geometrical validity of the structure. Based on the distinguishing results between the original dataset and newly generated ones, the network weights of both ***G*** and ***D*** are updated and frozen after sufficient iterations. A favorable generation accuracy for the four resonant frequencies can be obtained within the range without boundary outliers. Obviously, with the help of pixelated binary 2D images, a NN-based predictive model, and an outstanding generation strategy, high-accuracy design generations and experience-free design space explorations are demonstrated.

A bidirectional neural network is also used in the design of structural geometry. Cao et al. presented an approach for both forward performance prediction and inverse structural design based on a symmetric bidirectional neural network (SBNN) scheme, and a fiber optic vibration sensor was chosen as a design example [21]. A square diaphragm with various through holes (no holes in the center) is used to sense the acceleration, and its deflection triggered by accelerations will change the F-P cavity length to produce a corresponding output signal. Determining the diaphragm size and arranging the holes is the core task in the design work. The hole parameters are defined by a two-dimensional 10 × 10 2D matrix, where the position of numbers represents the location, and the magnitude represents the diameter (three for no holes and zero to two for holes defined by preset control strategies). In the forward prediction, a convolutional neural network (CNN) is firstly exploited to splice together the design parameters (namely 2D matrix, length, and thickness of diaphragm) and unidimensional performance parameters (characteristic frequency, sensitivity, and accompanying structural stress), whose dataset is built by simulating 10,000 randomly generated diaphragms. Then, the correlation between the two groups of parameters is mapped by an MLP-NN. In the structural design phase, the model above is symmetrically reversed to produce the inverse design network. In order to avoid the one-to-many problem, the inverse and forward networks are sequentially connected to form the SBNN. In the training, a tandem strategy is used, and the loss function is set as the mean square error between the initially input performances and forward predicted performances. The network can produce a suitable structure in 7.1 s with an accuracy of 96.2%, which saves 21.1× computational time compared to FEA methods.

### 3.3. Discussions on the Design Works

The NN methods have achieved favorable results in the generation of dimension and geometry for MEMS sensors, and the obtained structural schemes can fulfill the preset performances. These works often begin with the establishment of a labeled dataset through FEA, and the NNs are then trained to adapt the correlations between structure and performances. The available solutions are then produced by optimization algorithms or inverse networks. However, there are still several issues with the use of NN methods in designing MEMS sensors.

(1) The design work requires a labeled dataset that correlates the structural parameters and performance parameters. Currently, the available datasets are built through extensive finite element simulations. This process consumes a significant number of computational time and resources. At the same time, the coverage of the dataset is also affected by the simulation models, which may make the network trapped in local optimal solutions and lose its convergence. To improve the coverage, Sui et al. transfer the ability of humans to learn from a trial-and-error process to intelligent structural design [74]. After the establishment of performance predictors with supervised learning, deep neural networks are leveraged to constitute new designs to explore the design space efficiently through a step-by-step, trial-and-error manner, achieving a high generation accuracy of 97.7% based on prespecified targeted properties. However, there is still no appropriate method to address the issues in the construction of a FEA-based dataset, and further foundational investigation is needed.

(2) The treated parameters are limited. As the involved parameters increase, the workload from FEA also significantly increases. It is necessary to define the structure with fewer parameters, and the 2D-pixel models are utilized. The NN-generated structures often feature a dispersed, varied geometry and constant thickness. Inherently, the generation of more diverse configurations is still hindered by the limitation of treating more parameters.

(3) About the generalization of neural networks. Current design works usually focus on a specific object, and the specialization increases synchronously with the design results [75]. When the object changes, the entire process of modeling, FEA, and network training needs to be re-executed. More substantial datasets and innovated processing methods are urgently needed to achieve the more powerful AIGC-like tools.

## 4. NN Methods in the Fabrication of MEMS Sensors

The fabrication of MEMS sensors mainly involves determining the processes and relevant parameters, producing the mask layouts, chip micromachining, checking, and packaging. The established procedures and accumulated experiences in various institutions provide significant support for manufacturing. Inspired by the achievements of AI, several researchers are attempting to address the issues in sensor fabrication with neural network-based methods.

### 4.1. NN Methods in the Determination of Mask Layout and Fabrication Process

In the production of mask layout and fabrication process, Erik K. Antonsson’s group introduces the evolutionary algorithms to automatically fulfill this task [76,77]. A randomly generated initial mask layout and process flow are input into the simulation software, and the obtained processing pattern is then compared with the expected one. The parameters in GA are updated according to the deviations until the simulated results well match the expected structures. This approach has demonstrated its precision in the fabrication of convex corner structures in backside wet etching. Subsequently, the surface processes in MEMS devices are treated. The substrate, structural layer, and scarifying layer are determined according to the desired device, and the mask layout and fabrication process are then generated with the previous method [78,79,80]. Considering the common features in this work, some dedicated design tools, such as PROMENADE, have been developed and productized [81]. However, most of these mentioned works were finished before 2010, and the used proposals have not yet capitalized on the recent advancements in NN technology.

### 4.2. NN Methods in the Detection of Fabrication Defects

**Figure 6:**
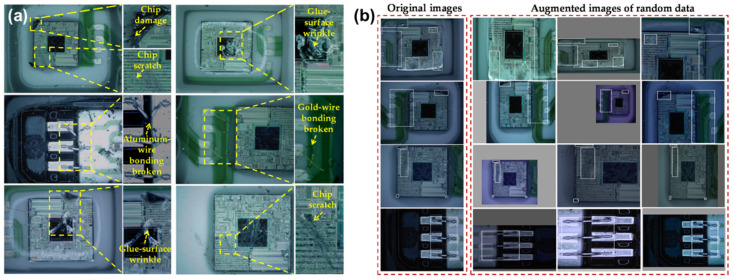
The image dataset for the defect detection in pressure sensors: (**a**) five kinds of defect images; (**b**) the data from augmentation. Reproduced under the terms and conditions of the Creative Commons Attribution License of [82].

The ability to accurately detect possible defects plays an essential role in the reliability evaluation of MEMS sensors. Along with the utilization of NN methods in pictures, defect detection in the micromachined wafers or packaged chips is also gradually conducted. Deng et al. proposed a CNN-based method for detecting the defects in the packaging process of MEMS pressure sensors [82]. The training dataset contained pictures with five kinds of defects, including chip scratch, chip damage, glue-surface wrinkle, broken gold-wire bonding, and broken aluminum-wire bonding (Figure 6a). To address the issue of insufficient datasets and uneven distribution of different defects, random-data augmentation based on image scale, flip, and color gamut distortion was conducted (Figure 6b). The obtained mean average precision can be 92.4% for the mentioned defects. Concerning the situation where multiple defects coexist, Yoo et al. constructed a randomized general regression network [83] and applied a novel information gain-based splitter [84] to remove random noise and reduce model bias during training. The detection accuracy can reach 99.8% for the individual defects and 86.17% for the multiple ones. Many similar works have been reported in the following years, and several practical methods have been proposed to augment the insufficient datasets [85,86,87,88,89,90]. However, the mentioned defect detection is mostly oriented toward the ICs, and there is still an urgent demand on the work specifically focused on the three-dimensional defects in MEMS sensors.

**Figure 7:**
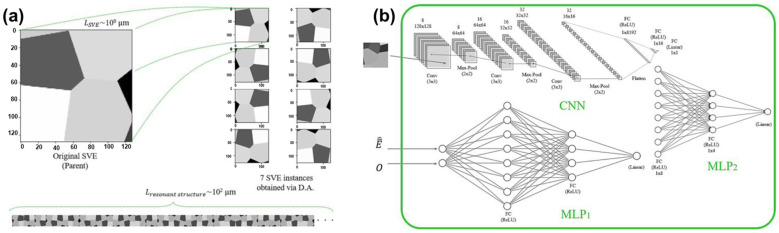
The two-scale multi-physics deep learning model for MEMS magnetometer: (**a**) Example of parent SVE, its instances, and the resonant structure; (**b**) architectures of the model used for the device-level mapping. Reproduced under the terms and conditions of the Creative Commons Attribution License of [95].

In device fabrication, the potential variations in material properties can also impact device performance. Employing suitable methods to ascertain the parameters of wafers is very helpful in ensuring the performance of sensors. As shown in Figure 7, a two-scale multi-physics deep learning model is proposed by combining CNN and MLP to provide an accurate property-performance mapping for a polysilicon single-axis, resonant Lorentz force MEMS magnetometer [91,92]. The CNN is firstly utilized to develop a regression model for mapping the microscale features (e.g., topology and orientation of grains) with the macroscale mechanical properties (e.g., Young’s Modulus) in polysilicon [93,94]. A set of statistical volume elements (SVEs) is digitally generated and simulated for the labeled dataset, which is augmented by proper rotations and flips. Then, a device-level model is established based on the SVEs with a probability distribution of fabrication detects for predicting the maximum oscillation amplitude of the clamped-clamped beam in the magnetometer [95,96].

### 4.3. Brief Discussion

Although neural network methods have shown certain application potentials in the manufacturing of MEMS sensors, they have not yet become an essential approach. Possible reasons include: (1) the generation of mask layout and process flows is already supported by some commercial tools, and the results from NN also require researchers to review and modify, leading to insufficient motivation in practical research and engineering. (2) Defect detection is essentially an image feature extraction and recognition problem, and it mainly depends on conventional image-treating methods. The related technologies are already very mature, but the lack of sufficient valid datasets for MEMS sensors makes this work not attractive enough to the relevant industries. (3) The lack of effective datasets also limits the NN methods in predicting the impacts of materials and fabrication parameters, and the large cost of constructing the datasets further worsens this situation. The solutions for these issues require further development in MEMS manufacturing technology and effective data accumulation.

## 5. NN Methods in the Calibration and Compensation of MEMS Sensors

Compensation and calibration are two critical issues when processing the output of MEMS sensors. Compensation is a measure to address the fluctuation of sensor outputs due to various environmental interferences. Appropriate compensation can help to maintain the device’s stability and reliability under different conditions. In the meantime, calibration can establish a relationship between output signals and input measurements, which is an essential step before its application. The two aspects are also the most extensively involved areas for neural network methods, which fully leverage the advantage of NN in learning from a large amount of data.

### 5.1. NN Methods in Compensation

The output compensation mainly involves the removal of interference information from the output sequences. Due to the diversity and impact-pattern complexity, it is a significantly challenging work to accurately model them, making it quite difficult work to finish high-precision compensation with conventional polynomial fitting ways [97,98,99,100]. Considering the capacity of NNs in processing large-volume data and discovering hidden correlations, there have been a significant number of studies using NNs to compensate for the outputs from MEMS sensors.

Temperature drift is the most encountered issue in the compensation of MEMS sensors [101,102]. The work primarily relies on the experimental data collected at various temperatures, which is requisite for training neural networks to identify the affecting patterns of temperature and then remove its influences. Currently, the backpropagation neural network (BPNN) and long short-term memory (LSTM) are two representative models.

**Figure 8:**
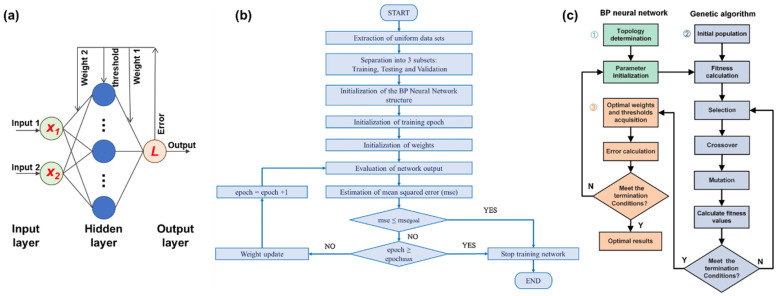
BPNN in the temperature compensation of MEMS sensors: (**a**) basic BPNN, (**b**) procedure for using BPNN, and (**c**) procedure for using GA-BP. Reused with the permissions from [106,108].

BP neural network is a widely used multi-layer feedforward neural network that has a strong adaptive capability to deal with complex, high-no-linear issues. The schematic diagram for a basic BPNN is shown in Figure 8a, and more information about its principle can be found in [103]. The typical procedure for using the BPNN in temperature compensation can be found in Figure 8b. Zhang et al. utilize a three-layered BPNN in the temperature compensation of a MEMS gyroscope, in which the network is trained with a dataset of three signal groups with time-domain outputs and corresponding temperatures. In the tested temperature of −20–20 °C, the forecast error variance for three axes is decreased from 0.424, 0.3506, and 0.4335 (using the conventional least squares method) to 0.0758, 0.1024, and 0.1122, respectively [104]. In the meantime, Fontanella et al. compensate for the drift of a three-axis gyroscope bias at the range of −24.4 °C to +40.8 °C and evaluate the advantages of the BPNN method using the quaternion approach. The application of the BPNN renders the MEMS gyroscopes with a longer time for keeping the flight attitude accuracy above the requirements of current regulations [105,106]. A similar scheme is also applied in the MEMS mass flow sensor [107]. Since the sensor measures the flow rate according to the temperature changes detected by internal thermopiles, its output value is inevitably affected by variations in ambient temperature [42]. Consequently, the authors input the testing results of 11 different flow rates at seven ambient temperatures to the BPNN, and a non-linear mapping relationship between temperature and flow rate can be established for compensation. Within the temperature of 0–60 °C, the relative error is stabilized at 0.2558% after BPNN compensation, compared to a maximum of 1.1016% after conventional polynomial fitting (the feature also continually varies with temperature). These applications demonstrate the superior performance of the BPNN in characterizing the impact of temperature drift and achieving temperature compensation.

However, the application of the BPNN in temperature compensation still faces several issues that need to be addressed. Firstly, the computation and data storage of the BPNN require sufficient resources, making it challenging to deploy in low-resource mobile embedded systems. Researchers have attempted to employ the B-spline method to fit the model of a trained BP neural network [109]. According to the evaluating results, the fitting method has reduced both computation time and data volume by more than 50%, and its compensation performance still surpasses those of polynomial fitting and regression tree methods. Then, the BPNN often suffers from its low learning rate and susceptibility to falling into local minimum [110]. Many efforts have been devoted to optimizing the initial parameters of the BPNN across a broader range. GA is among the commonly utilized methods [111]. Figure 8c shows a typical flowchart for the GA-BP in the sensor temperature compensation. After setting the structure of the BPNN, GA acts to globally optimize the weight and threshold of the BPNN before the training phase. This approach has demonstrated its superior feasibility in many MEMS sensors, such as resonant accelerometers [108], capacitive accelerometers [112], immune microwave sensors [113], and piezoelectric weight sensors [114], realizing a higher convergence rate and compensation accuracy. In the meantime, some variant searching algorithms also have played desired roles in initializing the parameters of the BPNN [115,116,117,118]. Moreover, data-cleaning methods (e.g., improved isolation forest algorithm) can help to remove the outliers in the dataset and prevent possible influences from erroneous data [116].

**Figure 9:**
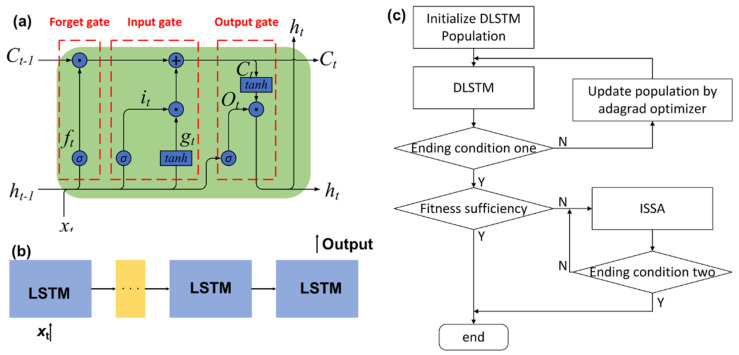
LSTM in the temperature compensation of MEMS sensors: (**a**) basic LSTM, (**b**) varied DLSTM, and (**c**) procedure for using ISSA-LSTM. Reproduced under the terms and conditions of the Creative Commons Attribution License of [121].

Although the BPNN has achieved good compensation performances in various sensors, the necessity of temperature information as one input parameter is not weakened. Meanwhile, the temperature sensor cannot be installed on every occasion because of the limitations of volume, technique, and cost. It is necessary to further strengthen the processing capacity to compensate for the temperature drift without knowing the temperatures. In the sensor outputs, the temperature interference and measurand-induced component are time-domain signals, and their variation tendency can indicate the information about temperature changes. In other words, it is possible to find an appropriate method to characterize the impact of temperature if the interference can be identified. As a continuous neural network, the LSTM provides an available solution for this situation. The LSTM network is a variant of RNN, which solves the problems of gradient disappearance and gradient explosion when the RNN learns long sequences [119,120]. The LSTM has an advantage in learning the time series volatility. Due to its unique design structure, the LSTM is suitable for processing and predicting the sensor output in time series without information on temperature interferences. A simple illustration of using the LSTM in the temperature compensation of MEMS sensors is shown in Figure 9.

Concerning the non-temperature circumstances, Cao et al. directly use the time-domain outputs of fiber optic gyroscope (FOG) as a dataset for the LSTM to complete the regression prediction of temperature error and eliminate the dependency on the temperature sensor [122]. The output signals under heating, holding, and cooling states endow the LSTM with a sufficient dataset. Compared with the other methods using temperature information for prediction and compensation, the LSTM-based method achieves obvious improvements in bias stability and Allan noise and reduces the complexity of the compensation system. Similar to the BPNN, the temperature can also be a participant in the LSTM. For instance, Jiang et al. utilize the normalized temperature vector as an input for the LSTM to compensate for the temperature drift in the scale factor and bias of FOG [123]. Moreover, the researchers attempt to introduce other algorithms, e.g., convolutional neural networks, support vector machines, and sparrow search algorithm, into the LSTM to achieve better noise classification and compensation [121,124,125,126,127].

In the output compensation of MEMS sensors, many neural networks are also employed to treat random drift, humidity-induced drift, noise reduction, and system errors [128,129,130,131,132,133,134,135,136]. Table 1 proposes a simple summary of the NN methods in the compensation of MEMS sensors. Regardless of the used models, the compensation is basically achieved by identifying the influence pattern of interferences on the key performances (mainly bias and scale factor) of devices and then removing the interferences that conform to this pattern. Additionally, the network training can be optimized by introducing other optimizing algorithms to enhance the learning speed. The whole compensation still relies on a vast amount of characterization results from extensive experiments, which can be a burden in practical application and desiderates further explorations.

### 5.2. NN Methods in Calibration

The input to a sensor is the to-be-measured parameter in the time domain, and the output is a corresponding modulated signal. Sensor calibration is the process that discovers the correlation between the sensor input and output, making it convenient for users to directly obtain the measurand information from the captured output. However, there is often nonlinearity and complex patterns in the correlation, and the possible coupling induced by multiple inputs can make the situation more serious. Therefore, conventional methods, e.g., polynomial fitting, often fail to yield satisfactory calibration results. Therefore, the NN method is frequently employed in the calibration of MEMS sensors to address the complex correlations in large-scale data. Generally, the calibration process can be divided into two main categories: inferring the measurand parameters and identifying/classifying the measurand. In the network training, the sensor outputs usually serve as the network input, while the expectant measured information can be obtained from the predicted output of the network.

#### 5.2.1. Inferring the Measurand Parameters

**Figure 10:**
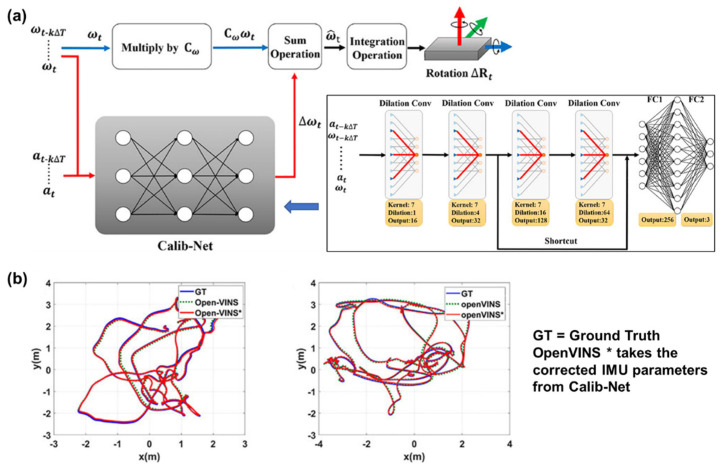
Calib-Net for low-cost IMU calibration: (**a**) the overview of calibration process; (**b**) calibration performance evaluation using VIO methods. Reproduced under the terms and conditions of the Creative Commons Attribution License of [139].

MEMS IMUs are cost-effective, self-contained, small-size, low-power devices used in various motion tracking and sensing applications. However, the relatively low accuracy and stability greatly hinder their actions on high-end occasions. It is necessary to propose a high-performance calibrating method to adopt the precision inertial parameters from these low-grade output signals, where NNs are greatly involved [137,138]. There are two examples to show the advantages of utilizing NNs in the calibration of MEMS IMUs. Firstly, the calibration can be directly conducted based on the original sensor outputs. Li et al. proposed a lightweight and efficient deep convolutional neural network (DCNN), called Calib-Net, for low-cost IMU calibration [139]. The temporal measurements of the gyroscope and accelerometer are taken as the inputs, and the trained Calib-Net can output a corresponding compensation part for angular velocity measurements, which will be used to improve the test quality of angular velocity based on the intrinsic calibration matrix of IMU (Figure 10). This scheme shows an obvious promotion in orientation estimation and is comparable with the state-of-the-art visual inertial odometry methods. Then, the measurements of high-grade IMUs can be utilized as a participant in the calibration work. Mahdi et al. propose an adaptive neuro-fuzzy inference system (ANFIS), a technique that fusing the ANN and fuzzy inference system, to leverage the performance of low-grade IMUs. In this work, the ANFIS is trained with the high-end IMU signals as output and the low-grade IMU measurements as input. The results show that the RMSE of IMU signals is obviously decreased by the new method, and an improvement of 70% in the 2D positioning and of 92% in the 2D velocity of the INS solution were attained when compared with the value from raw data. The NN-based method also plays its role in the calibration of single MEMS accelerometers. Soriano et al. input the test values of a two-axis accelerometer to the neural network, and the actual loaded accelerations are used as outputs in the training. The nonlinearity is corrected, and measuring characteristics are successfully calibrated. The results showed that 90% of the sensor’s nonlinearity is corrected, and the maximum error in testing is reduced by 99% [140].

**Figure 11:**
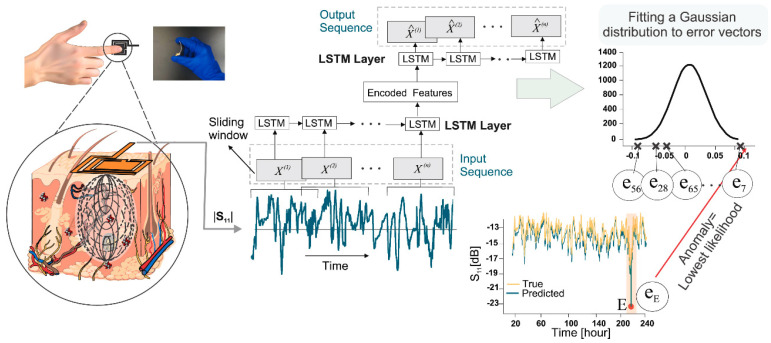
The learning framework of using the LSTM model in human glucose monitoring. Reused with the permission from [144].

Similar calibration methods can also be used to determine the concentration of material under test (MUT) by measuring the dielectric constant with microwave sensors [113]. The working principle of these devices can be found in the relevant references [141,142]. Chaisaeng et al. use the soluble solid content (SSC) of a sugar solution and the corresponding intermediate frequency of an X-band sensor as the dataset to train an ANN. A predicting accuracy of 92.98% for the SSC is achieved, endowing the low-cost X-band sensor with the ability of classifying SSC level determination in real-time [143]. A similar approach can be found in the in-vivo test for non-invasive monitoring of glucose in humans [144]. The whole process of this work is indicated in Figure 11. The |S11| of a coupled SRR/Patch under a fingertip press is used as the input of the LSTM to detect possible anomalies in blood GL and predict its future values. The resultant sensing system has demonstrated its great potential for being deployed in highly sensitive applications for noninvasive interrogation of ambient interstitial glucose levels. Albishi modulates the frequency response frequency responses of microwave near-field sensors based on a two-port microstrip line to characterize the material parameters [145]. A Bayesian regulation backpropagation artificial neural network (BRANN) produces a robust model to predict the dielectric constants of different MUTs (obtained by adjusting the ratio of chloroform and cyclohexane) through the frequency responses of sensor. The realized accuracy for analyzing MUT composition can be up to 99.97%.

#### 5.2.2. Material Identification

**Figure 12:**
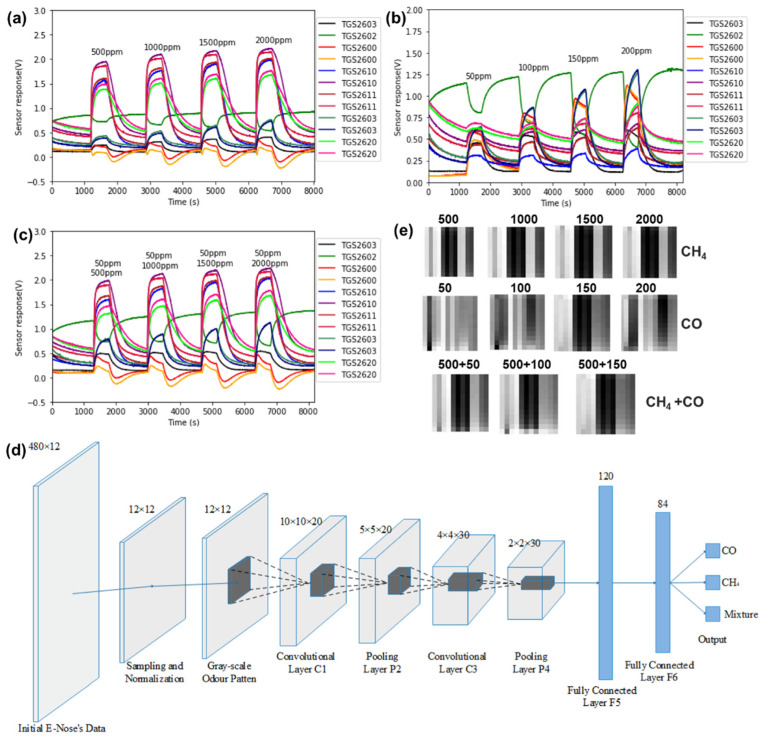
Using LeNet-5 for gas identification: the responses of 12 sensors to CH4 (**a**), CO (**b**), and CH4 + CO (**c**); (**d**) the proposed LeNet-5 structure for gas identification; (**e**) patterns of downsampled matrixes used in LeNet-5 for different gases (unit: ppm). Reproduced under the terms and conditions of the Creative Commons Attribution License of [151].

Testing and recognizing the appeared gases is a common task for material identification [31,146,147]. The highly selective detection can be achieved by leveraging the distinct influence patterns of gases on a single sensor or array [32,148,149,150]. Figure 12a–c indicates the response curves of different gases from 12 commercial metal oxide gas sensors [151]. It can be observed that the responses evidently vary with the gas composition and concentration, which provides a strong marker for the identification. Consequently, the authors employed the LeNet-5, a gradient-based learning structure, to identify three gases, namely pure CO, pure CH_4_, and mixtures of CO and CH_4_ (Figure 12d). A response matrix (480 × 12) **X** = ***x***_i,j_ is proposed to show the normalized response values of the 12 sensors (sensor number is indicated by subscript j) over a 480-s time period (time is indicated by subscript i). Figure 12e shows some typical patterns (12 × 12) of CH4, CO, and gas mixtures obtained through downsampling the matrix with an interval of 40. Each preprocessed grayscale pattern represents the information of the sensor array corresponding to the test analyte. After dataset augmentation with translation and cropping, the obtained 1000 matrixes are input to the LeNet-5. After a 12.73s training period, the model can achieve an identification accuracy of 99.67% within a time frame of 0.01553 s. Although the training time is slightly longer than conventional methods (e.g., MLP and SVM), the recognition accuracy has been significantly improved (enhanced by 10% at least), while the testing time shows no significant change. Similarly, Peng et al. use the DCNN in treating the responses of electronic noses to realize a high-performance detection of carbon monoxide, methane, hydrogen, and ethylene, and the accuracy is also higher than simple network models [152]. These works obviously distinguish the superiority of deep networks in gas identification. One of the possible reasons is that the sensor responses can be interfered using ambient noises, which may bring about false features if the treating capacity is limited by the simple models.

**Figure 13:**
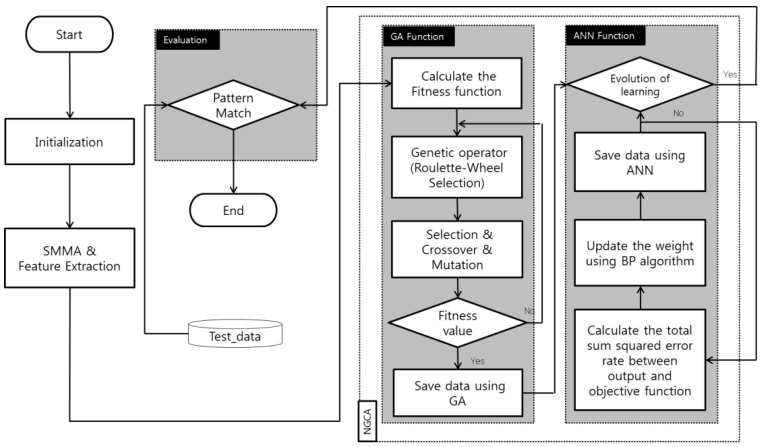
Flow of the gas recognition with SMMA for denoising, GA for data extraction, and BPNN for recognition. Reproduced under the terms and conditions of the Creative Commons Attribution License of [153].

In the meantime, some attempts have been devoted to the data preprocessing before network training. For example, Kim et al. propose an identification module in Figure 13 [153]. They preprocess the signal with the smoothed moving average (SMMA) to facilitate data smoothing, noise elimination, and filtering. Then, GA adopts the data with high fitness from the received ones, and the generated gradient is passed to the BPNN for odor detection. The developed recognition module can extract patterns with greater reliability and then realize a high success rate in detecting several gases. Moreover, the fuzzy neural network has proved to be a powerful tool in identifying the MUT through high-noise data [154]. The accurate MUT identification offers a dependable benchmark for devising the operational strategies of environmental monitoring nodes, enabling the timely optimization of data acquisition precision and transmission volume for better energy efficiency [155]. These works will be of meaningful assistance to IoT-compatible device technologies.

#### 5.2.3. Data Supplement in the Calibration

The datasets in the abovementioned works are all based on the measured responses, and their establishment often requires substantial time and manpower/material resources. As mentioned in [151], the acquirement of each response curve requires 28 min for sensing with extra time for chamber cleaning and a three-day warmup at the beginning of each experiment batch. Coupled with the extensive data required by neural networks, this poses a considerable challenge for research endeavors. Therefore, researchers have attempted to find a theoretical or simulation model to preliminarily create the input–output data, which can be the footstone for training datasets [156]. The experimentally obtained data will only serve as a medium for validating the invented models in the transfer learning ways, whose essential volume can be greatly decreased.

**Figure 14:**
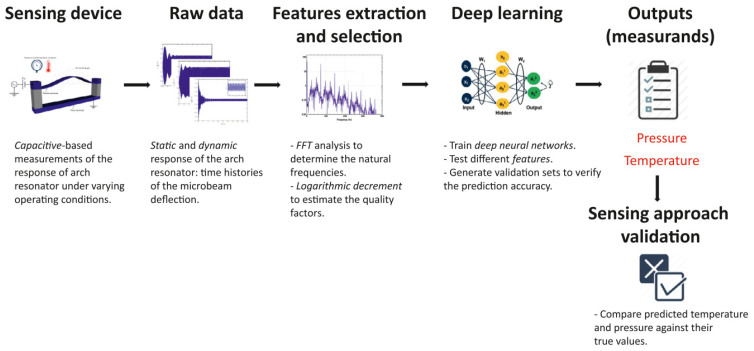
Block diagram illustrating the sensing approach that combines physics-based modeling and deep learning. Reused with the permission from [157].

Ghommem et al. conducted a series of meritorious works investigating the features of cantilever sensors with a scheme combining physics-based modeling and deep learning, which successfully solves the issues during calibrating flow, temperature, and gas sensors. For instance, Figure 14 shows a process of using this method in a pressure-temperature sensor [157]. The static and dynamic responses of arch resonators under different conditions are firstly simulated by the physics physics-based model to generate the raw data. Necessary feature extraction and selection are conducted, and the obtained features (resonant frequency and quality factor here) are passed to the fully connected neural networks for training. Then, the trained network can output the predicted pressure and temperature. Before training, the sensor responses from the physics-based model should be validated by the existing experimental results to ensure its accuracy. Similar pre-validation with experimental results also appears in the measurements of flow density/viscosity, gas concentration, and nonlinearity of resonant cantilever [158,159,160,161].

The response model form simulation is another available source for data supplement [162]. A full MEMS-ASIC system for a capacitive accelerometer is modeled in Matlab/Simulink to provide the required dataset for NN. Then, the experimental results of the known device are transferred to the NN for fine-tuning parameters. Thus, the fine-tuned NN can predict the core parameters of sensors based on a limited amount of experimental data. This work can further facilitate the inspection of wafer-level MEMS devices. In the meantime, a high-frequency full-wave solver based on FEM (e.g., HFSS) is used to capture the transmission response of a complementary split-ring resonator (CCSRR) sensor underwater, ethanol, methanol, and their mixtures. The obtained frequency, amplitude, and quality factor are input to DNN/CNN for predicting concentrations [163]. Furthermore, the sensing features predicted by NN in the design phase and augmented data from generative adversarial networks can also be used to supplement the dataset to promote sensor calibration [164,165].

### 5.3. Brief Summary and Discussion

Both compensation and calibration focus on the processing of sensor outputs, which complement each other in the development of high-performance MEMS sensors. In summary, applying neural network-based methods to this domain may involve the following three aspects:

(1) NNs have played prominent roles in the sensor compensation. This operation can provide stable and reliable data for characterizing the measurand, and the deviations of the sensor at bias and operation states have been well treated. It is worth noting that the drift in the scale factor can only be compensated after obtaining the bias drift and operation data.

(2) The main target of output processing focuses on the establishment of a correct and reliable correlation between measured parameters and sensor outputs. It is better to conduct the calibration after necessary compensation, but the additional workloads may impede this procedure. Therefore, it is more practical to comprehensively consider the data amount, consumed resources, and target measuring performances when determining the processing method.

(3) A desired scheme for compensating unknown interferences in a new environment is using the pre-calibrated model as a reference to assess the interference situation, which can simplify the reorganization of the interfering pattern and further accelerate the secondary compensation. However, the published papers mainly focus on compensation and calibration under known interferences. The trial for unknown interferences is still waiting for further investment.

Meanwhile, NN heavily relies on the volume of obtained experimental data. Although some theoretical and simulation predictive models can be a supplement, the establishment of these models still requires additional work. The quality of these generated data may suffer from the coverage and accuracy. These issues are still awaiting a more convenient solution to produce supplemental data that closely align with actual experimental results.

## 6. Challenges

Although many milestones have been achieved, NN-based methods in the development of MEMS sensors still require great devotion. Its progress journey may face the following challenges:

(1) More comprehensive development. Current NN-based methods only focus on one aspect of sensor development, and a more comprehensive NN model to cover the interfaces between design, fabrication, and device testing is still urgently needed. Furthermore, the measuring characteristics of sensors are often indirectly assessed using several intermediate parameters, such as structural deformation, stress/strain, and resonant frequency. There is a significant gap between prototype experimental results and design parameters that hinders comprehensive development in this domain. These challenges require more effective modeling and design methods (e.g., combining NNs and the Design Technology Co-Optimization concept in the semiconductor industry), as well as advanced training approaches, to support the comprehensive development of MEMS sensors.

(2) The dataset generation. The data used to train neural networks in current developments mainly comes from FEA and actual experiments. The data generation consumes a significant amount of manpower/material resources and takes a lot of time to fulfill this task. Although the efficiency of trained NNs is significantly higher than traditional methods, the dataset generation still reduces the development cost and cycle for MEMS sensors. Physical models may be an available data supplement, but the involved multiple parameters make the establishment a highly professional task. The accuracy of proposed models across the entire design space also significantly affects the prediction capacity of NNs. More appropriate methods that better combine theoretical models, simulation models, and experimental data could be helpful in generating effective datasets and accelerating the development process.

(3) Universal evaluation for network performance. This paper shows that neural networks have exhibited the desired performance in various MEMS sensors. However, the execution of each task often depends on self-built datasets, and the performance evaluation often relies on the comparison with traditional solutions. The lack of a universal benchmark makes it challenging to compare the performances of different reported network models. The application of NN in image, video, text, and speech has accumulated a wealth of general datasets for evaluating different models. The research on NNs in MEMS sensors is still in an early stage, and the diversity of investigated sensors also causes great difficulties in this issue. The establishment of general datasets for universally evaluating the networks in MEMS sensors still requires significant manpower and long-term commitment.

(4) Generalization of neural network models. Limited by the lack of a general dataset, the trained networks only exhibit well in addressing a certain problem in certain MEMS sensors and cannot migrate to other unseen ones with existing knowledges. It is challenging to achieve a balance between the capacity to address specific predicting demands and the versatility to adapt to a wide range of scenarios. Establishing appropriate datasets, optimizing network parameters, and achieving better network generalization will be an inevitable problem in the future. The recently developed Physics-Informed Neural Networks can encode the physical model equations as a component of the neural network and could be a potential solution for a desirable generalization [166].

## 7. Conclusions

The methods based on NNs have played a distinguished role in the development of MEMS sensors. As for the structural design, NN models have effectively described the relationship between structural parameters and device performance, enabling the optimization of key size parameters. Furthermore, some improved models, e.g., tandem networks, have been used to achieve viable structural generators. The device fabrication has also received certain assistance in material assessment and defect detection. When processing sensor outputs, neural networks have demonstrated their significant advantages. The BPNN can accurately recognize the patterns of temperature impact and compensate for the resulting drifts in bias and scale factor. The LSTM can even perform temperature compensation without the information from the temperature sensor. With appropriate data processing, NN models can calibrate the detection characteristics of various sensors to establish stable input–output relationships or identify the characteristics of MUTs. Obviously, neural network methods have brought about great innovation and a high tempo for the development of MEMS sensors. However, current research still faces challenges in comprehensive design capability, dataset generation, universal evaluation, and generalization, and continued efforts are needed to make NNs a more powerful game changer in the development of MEMS sensors.

## Figures and Tables

**Table 1:** Summary of the NN models in sensor compensation.

NN Model	Assisted Algorithm	Device	Dataset	Ref.
BPNN	-	Gyroscope	Bias and T	[104,105,106]
BPNN	-	Flow sensor	Output and T	[107]
BPNN	GA	Accelerometer	Output and T	[108]
BPNN	B-spline	Gyroscope	Bias and T	[109]
BPNN	AGA	Accelerometer	Output and T	[112]
BPNN	GA	Weight sensor	Output and T	[114]
BPNN	Cuckoo search	Pressure sensor	Output and T	[115]
BPNN	IIF and ISSA	Laser methane sensor	Output and T	[116]
BPNN	IFA	Accelerometer	Output and T	[117]
LSTM	-	FOG	Output	[122]
LSTM	-	FOG	Output and T	[123]
LSTM+CNN	PSO-SVM	Gyroscope	Bias and T	[124]
Deep LSTM	ISSA	Accelerometer	Bias and T	[121]
LSTM	SVM-DBN	Gyroscope	Output, T and T CR	[125]
LSTM	-	FOG	Output and T CR	[126]
GRNN	EEMD	Accelerometer	Bias with error	[130]
Deep BPNN	SGD	NO_2_ sensor	Output, T and H	[131]

Acronym list: AGA—adaptive genetic algorithm; IIF—improved isolation forest; ISSA—improved sparrow search algorithm; IFA—improved firefly algorithm; SVM-support vector machine; DBN—depth belief network; EEMD SGD—stochastic gradient descent; FOG—fiber optic gyroscope; T—temperature; CR-change rate; H-humidity.

## Data Availability

The original contributions presented in the study are included in the article, further inquiries can be directed to the corresponding author.
